# Routine postoperative blood tests fail to reliably predict procedure-related complications after laparoscopic cholecystectomy

**DOI:** 10.1007/s00423-021-02115-x

**Published:** 2021-03-24

**Authors:** Jens Strohäker, Lisa Wiegand, Christian Beltzer, Alfred Königsrainer, Ruth Ladurner, Robert Bachmann

**Affiliations:** Department of General, Visceral and Transplantation Surgery, University Hospital of Tuebingen, Hoppe-Seyler-Straße 3, 72076 Tuebingen, Germany

**Keywords:** Laparoscopic cholecystectomy, Perioperative care, Gallstone disease, Biliary complications, Blood tests

## Abstract

**Purpose:**

Laparoscopic cholecystectomy is a highly standardized surgical procedure with a low risk of complications. However, once complications develop, they can be life-threatening. The aim of this study was to evaluate the value of blood tests on postoperative day one regarding their potential to predict postoperative complications

**Methods:**

A cohort study of 1706 consecutive cholecystectomies performed at a tertiary hospital and teaching facility over a 5-year period between 2014 and 2019.

**Results:**

Patients that had open CCE or conversion CCE were excluded. One thousand five hundred eighty-six patients were included in the final analysis that received a laparoscopic cholecystectomy (CCE). One thousand five hundred twenty-three patients had blood tests on POD 1. Forty-one complications were detected including 14 bile leaks, 2 common bile duct injuries, 13 choledocholithiasis, 9 hematomas, and 2 active bleedings. Bilirubin was elevated in 351 patients on POD 1. A drop of more than 3 mg/dl of hemoglobin was reported in 39 patients. GPT was elevated 3 × above the upper limit in 102 patients. All three tests showed a low sensitivity and specificity in detecting postoperative complications.

**Conclusions:**

Early postoperative blood tests alone show a low specificity in detecting postoperative complications after laparoscopic CCE. Their main benefit appears to be the negative predictive value, when they are normal. Routine blood testing appears to be unnecessary and should be based on the intraoperative diagnosis and postoperative clinical findings.

## Introduction

Cholecystectomies (CCE) are among the most common surgical procedures in general surgery departments. In 2017 over 200.000 cholecystectomies were performed in Germany, the vast majority being done laparoscopically [1]. Complications are rare but may be life-threatening to the patient [2, 3]. They are detected less frequently when CCE is performed for polyps or chronic cholecystitis. When CCE is performed for acute cholecystitis, complications may present in up to 10% of patients [2]. In order to detect complications early, most hospitals perform routine postoperative blood tests and ultrasound [4]. However, little data exists on the true benefits of post-op labs and their capability to predict complications. Centers that run cholecystectomy as day cases, long for better tools to decide who to admit to the ward and who to discharge postoperatively [5]. Our perioperative protocol consists of two routine blood tests. Preoperative labs include complete blood count (CBC), international normalized ratio (INR), prothrombin time (PTT), total bilirubin, and glutamate-pyruvate-transaminase (GPT) / alanine-amino-transferase (ALT). Patients are usually admitted on the day of surgery, then have repeat labs on the morning of postoperative day one (POD 1), and are discharged on postoperative day two (POD 2). We used to perform routine postoperative ultrasound for every patient, but abandoned to do so after detecting a high rate of false positive findings, while missing out on true complications [6]. Elevated liver function tests preoperative are considered to be the most reliable tests in order to predict present stones in the common bile duct. [7]. To our knowledge, there is still very limited data on the predictive value of blood tests on POD 1. Thus, the aims of our study were to evaluate the value of routine postoperative blood tests and their capability to detect procedure-related complications.

## Materials and methods

The Department of General, Visceral and Transplantation Surgery of the University Hospital of Tuebingen, Germany, serves two purposes. While it is an academic tertiary care teaching facility, it is also the sole provider of (acute) inpatient surgical and medical care for the district of Tuebingen, Germany. We retrospectively analyzed the data of all consecutive cholecystectomies that were performed at our hospital as an independent procedure from 2014 to mid 2019. Overall a total of 1706 cases were identified from the clinical database. We excluded 66 patients that underwent scheduled open cholecystectomy as well as 54 patients that had a conversion from a laparoscopic to an open procedure. The overall conversion rate was 3.2%. Nearly half of the cases were done by general surgery residents. Due to teaching aspects, CCE is performed with the electrocautery hook instrument in a standard four-trocar technique. We do not perform routine intraoperative cholangiogram. When patients present to the hospital with symptoms and/or imaging suspicious of choledocholithiasis (CDL), our standard is to perform endoscopic retrograde cholangiography (ERC) prior to surgery. Once CDL has been cleared and post-ERC pancreatitis is ruled out, we perform cholecystectomy. Our surgical endoscopy department attempts ERC even in patients that have undergone upper gastrointestinal surgery (e.g., Billroth II or bariatric gastro-jejunal bypass). If ERC fails to resolve CDL, intraoperative cholangiography is performed.

The intraoperative placement of drains is done at the surgeon’s discretion. During all procedures, a board certified surgeon is present throughout the whole case.

Non-emergent cases are admitted in the morning, undergo the procedure on that day, and are scheduled to receive post-op labs on the morning of POD 1 in order to be discharged the following day. Blood tests on POD 1 consist of a complete blood count (CBC) as well as GPT/ALT and total bilirubin. We do not routinely measure postoperative c-reactive protein in non-emergent cases. It is done at the staffs’ discretion based on intraoperative findings and postoperative course. When the staff deemed necessary, additional parameters were collected or labs were repeated on POD 2. Our hospital is the sole provider of cholecystectomy and ERC for the district of Tuebingen. Procedure-related complications were defined as all complications that were documented in the in-house charts within 90 days of the procedure and were classified according to Dindo-Clavien [8]. For the purpose of analysis, we assumed that (nearly) all patients would present or be referred to our facility with postoperative problems. We additionally screened all records regarding the need for ECRP even later than 90 days after CCE. We labeled all complications as either bleeding related, bile duct related, or infection related.

Comparison between groups was carried out by Chi-Square test or Fisher’s exact test for nominal variables and Mann–Whitney *U*-test or Kruskal–Wallis test for continuous variables, as appropriate. A probability of less than 0.05 was considered to be statistically significant. All *p* values reported are results of two-sided testing. Where needed, Bonferroni-correction was applied. Statistical analysis was carried out using IBM SPSS Statistics for Windows, Version 26.0 (IBM Corp., Armonk, NY, USA). We calculated the sensitivity, specificity, as well as positive and negative predictive values for the laboratory tests.

## Results

A total of 1586 consecutive patients that underwent laparoscopic cholecystectomy were included in the final analysis. The patient characteristics are shown in Table 1. Overall, 41 procedure-related complications developed in 40 patients (2.5%), and the reoperation rate was 10 (0.6%). Four patients were diagnosed with simultaneous choledocholithiasis and biliary leak postoperatively. The readmission rate was 7 (0.4%). Four patients were readmitted for choledocholithiasis and three for bile leaks. For details, see Table 2. Scheduled blood tests were performed on the morning of the first postoperative day in 1523 patients (96%) patients. A total of 331 patients (21%) had repeat blood tests on POD 2, and 50 patients (3%) had blood tests only on POD 2.

**Table 1:** Shows the patient characteristics of the included patient cohort

Patient characteristics (*n* = 1586)		
Gender m/f	588/998	37%/63%
Median age in years	54	Range 16–97
BMI	27	Range 17–60
Intraoperative diagnosis		
Acute cholecystitis	353	22.3%
Chronic cholecystitis	270	17.0%
Symptomatic cholecystolithiasis	920	58.0%
Tumor	43	2.7%
Emergency cases	261	16.5%
Median operating time in minutes	69	Range 16–302
Median length of stay in days	2	Range 1–51
ERCP findings		
Preoperative (*n* = 240)		
Sludge	45	2.8%
Choledocholithiasis	150	9.5%
No pathologic findings	45	2.9%
Postoperative (*n* = 41)		
Choledocholithiasis	14	0.9%
Bile leak	14	0.9%
Benign stenosis	1	0.1%
No pathologic findings	12	0.8%

**Table 2:** Shows patient characteristics as well as pre- and postoperative ERCP findings and procedure-related complications

Complications 90 days		%
Complications overall	*n* = 41	2.6
Bleeding related		
Acute bleeding	2	0.1
Hematoma	9	0.6
Bile related		
Bile leak	14	0.9
Cystic duct insufficiency (*n* = 9)		
Common bile duct lesion (*n* = 3)		
Accessory bile duct (*n* = 1)		
Duodenal perforation (*n* = 1)		
Choledocholithiasis^1^	13	0.8
Occlusion of common bile duct	2	0.1
Thrombosis ascending to IVC	1	0.1
Reoperation	10	0.6
Bile leak from cystic duct	1	0.1
Active bleeding	1	0.1
Hematoma	3	0.2
Impacted gallstone	1	0.1
Common bile duct injury	2	0.1
Perforated duodenal ulcer^2^	1	0.1
No pathologic findings	1	0.1
Death	3	0.2

^1^The discrepancy between the numbers in post-op ERCP findings and complications of post-op CDL is due to known but not removable preexisting gallstones

^2^A perforated duodenal ulcer was the source of one bile leak

### Hemoglobin and bleeding complications

Two patients suffered from acute bleeding, nine developed hematoma in the former gallbladder bed or intra-abdominally, but without signs of acute bleeding when the complication was discovered. The median pre-op hemoglobin was at 13.6 mg/dl. Median decrease in hemoglobin between pre-Op and POD 1 was 1.0 mg/dl. There was no difference between the patients that developed bleeding complications and those who did not (median drop of hemoglobin, 1.5 mg/dl vs 1.0 mg/dl, SD ± 1.9 mg/dl vs ± 1.0 mg/dl, *p* = 0.39). We performed an ROC analysis (not shown) to find the best sensitivity and specificity for a hemoglobin drop. The recommended value was a decrease of 2 mg/dl. The sensitivity was 50%, while the specificity was 84.7% with a false-positive rate of 14.3%. We decided to sacrifice sensitivity to increase specificity for this analysis and therefore chose 3 mg/dl as our cutoff. This decreased the sensitivity to 37.5% with an increase of the specificity of 97.6%. The negative predictive value was 99.6%. Forty-three patients (2.8%) had a decrease in hemoglobin of 3.0 mg/dl during the same timespan. Two patients developed acute bleeding from the cystic artery, while nine developed self-limiting gallbladder bed hematoma. The two patients with acute bleeding were not discovered via routine blood tests on POD 1 but rather deteriorated clinically (tachycardia and hypotension). Most of the hematomas were discovered during ultrasound examinations, of which three were done for drop of hemoglobin, the rest were coincidental findings during ultrasound for hyperbilirubinemia or revision surgery. None of these had true clinical relevance (such as compression of the portal vein or vena cava) (Table 3).

**Table 3:** Schows sensitivity and specificity of postoperative hemoglobin drop with regards to bleeding complications, as well as hyperbilirubinemia as a predictor of CDL or CBD occlusion (surgical and / or stones)

Sensitivity and specificity of postoperative blood tests POD 1			
	Bleeding	No Bleeding	
Hemoglobin drop > = 3 mg/dl	3	36	Positive predictive value 7.7%
Hemoglobin drop < 3 mg/dl	5	1469	Negative predictive value 99.6%
	Sensitivity 37.5%	Specificity 97.6%	total n = 1513
	Choledocholithiasis	No Choledocholithiasis	
Abnormal bilirubin POD1	9	342	Positive predictive value 2.6%
Normal bilirubin POD 1	4	1140	Negative predictive value 99.7%
	Sensitivity 69.2%	Specificity 76.9%	total n = 1495
	CBD occlusion	No CBD occlusion	
Abnormal bilirubin POD1	10	341	Positive predictive value 2.8%
Normal bilirubin POD 1	5	1139	Negative predictive value 99.6%
	Sensitivity 66.6%	Specificity 77.0%	total n = 1495

### Hyperbilirubinemia and biliary complications

Fourteen patients developed biliary leaks, while thirteen patients were diagnosed with new choledocholithiasis within the study period. Two patients suffered from iatrogenic injury of the common bile duct. The median total bilirubin pre-operatively was 0.7 mg/dl. On POD 1, the median bilirubin was 0.8 mg/dl. In our collective, 295 patients (18.6%) had elevated bilirubin (> 1.1 mg/dl) preoperatively compared to 351 with an elevated bilirubin on POD 1 (22.1%). There is a positive correlation between preoperative and postoperative bilirubin (Spearman R 0.587 *p *< 0.001). Independently 109 patients with previously normal bilirubin developed new hyperbilirubinemia on POD1 compared to 171 patients with preoperative hyperbilirubinemia bilirubin whose bilirubin returned to normal on POD1.

The fourteen patients who developed a bile leak had a comparable preoperative white cell count to those who did not (preoperative median of 8515/µl vs 7390/µl, SD ± 7635/µl vs ± 4374/µl, *p* = 0.10). There was a statistically significant difference on POD 1 (median POD 1 of 11,315/µl vs 9190/µl, SD ± 8617/µl vs ± 3688/µl *p* = 0.03, *R* 0.05, low effect size). Preoperative and postoperative bilirubin was similar between patients developing bile leaks and their counterparts (preoperative median of 1.0 mg/dl vs 0.7 mg/dl, SD ± 0.7 mg/dl vs ± 0.8 mg/dl, *p* = 0.11) (median POD 1 of 0.8 mg/dl vs 0.8 mg/dl, SD ± 0.7 mg/dl vs ± 0.8 mg/dl, *p* = 0.55).

Of the thirteen patients that were later diagnosed with choledocholithiasis, six patients already suffered from preoperative hyperbilirubinemia, while on POD 1, nine of those thirteen patients had an elevated bilirubin. Hyperbilirubinemia on POD 1 has a sensitivity of 69.2%. The positive predictive value is low at 2.6%. The negative predictive value was 99.7%. The odds-ratio of being diagnosed with postoperative choledocholithiasis is 7.50 in the presence of hyperbilirubinemia on POD 1. The median time to diagnosis of postoperative CDL was 6 days (SD ± 2,8; range 2–9 days). For details, see Table 3.

We performed ROC analyses and compared preoperative and postoperative bilirubin levels with regard to new postoperative biliary obstruction (both due to stones or bile duct ligation). Preoperative bilirubin had an area under the curve (AUC) of 0.72 (CI 0.5–0.86, *p *= 0.005). Bilirubin on POD 1 had an AUC of 0.74 (CI 0.59–0.90, *p* = 0.002). However, when we eliminated all patients with abnormal preoperative bilirubin ROC showed an AUC of 0.61 for bilirubin on POD 1 (CI 0.37–0.85, *p* = 0.31).

A linear regression was performed to identify predictors of hyperbilirubinemia on POD 1. Preoperative hyperbilirubinemia (*r* = 0.79, *p *< 0.001), bile metabolism disorders (Gilbert’s disease) (*r* 0.25, *p* = 0.004), and liver cirrhosis (*r* 0.25, *p* < 0.001) were the strongest predictors of hyperbilirubinemia on POD 1 and thus may lead to false assumptions when detecting elevated bilirubin on POD 1.

### Detection of biliary complications

In 264 patients, a drain was placed at the surgeon’s discretion. The drain was scheduled to be removed either on POD1 or later depending on the quantity and quality of the fluids it drained. Most drains were placed in patients with acute cholecystitis (*n* = 151, 42.8%) followed by symptomatic cholecystolithiasis (*n* = 63, 2,5%), chronic cholecystitis (*n* = 47, 17.4%), and tumor (*n* = 3, 6.9%). Of the 14 patients that developed a bile leak, 11 had a drain placed intraoperatively. Nine of these bile leaks were diagnosed with the help of that drain. Of the remaining five patients, three were discharged and readmitted due to upper abdominal pain caused by bilioma. One leak was found on POD 1 during planned ERCP after the surgeon had seen a tear in the common bile duct (CBD) intraoperatively but abstained from trying to repair it. The last one was an incidental finding during post-op ERCP for stent removal.

Overall 255 drains were placed and removed without detecting a complication. The number of drains needed to be placed to detect a leak was 33. The intraoperative placement of a drain led to a longer length of stay. (median of 2 vs 3 days, SD ± 1.2 vs ± 4.7, *p* < 0.001, *R* 0.46, medium effect size). The same is true for a subgroup analysis of the non-emergent cases. Here 129 drains were placed with the same elongation of stay (median of 2 vs 3 days, ± 0.9 vs ± 2.4, *p* < 0.001, *R* 0.36, medium effect size).

Postoperative choledocholithiasis was detected thirteen times in our cohort. The most common trigger for further diagnostics was increasing bilirubin (*n* = 9) followed by clinical deterioration (*n* = 1). In three cases, the stones were detected during ERCP done for other reasons.

In our patient cohort, two major choledochal injuries occurred. Both were d3 injuries according to the Hanover classification of iatrogenic bile duct injuries[9] that were discovered due to a rise in serum bilirubin, one accompanied by biliary fluid in the drain. One was repaired sufficiently by hepatico-jejunostomy (HJ). The other one developed septicemia due to repeated leak following HJ and then succumbed to the complications. The overall procedure-related mortality was 0,06% (*n* = 1/1586).

### Transaminases

In our collective, no major vascular injuries occurred. GPT/ALT was collected from 1139 patients on POD 1 (71.8%). Median pre-op GPT/ALT was 28 U/l (SD ± 87 U/l range 4–681). On POD1, the median GPT/ALT rose to 51 U/l (SD ± 80 U/l range 8–1509). One hundred and two patients had a GPT/ALT greater than three times the upper limit of 50 U/l. Two patients had a GPT greater than 1000 U/l on POD1. There was no difference in GPT/ALT on POD 1 between patients that suffered from complications and those who did not (*p* = 0.13).

### Acute cholecystitis vs. elective cholecystectomy

We performed a subgroup analysis that compared the cases of acute cholecystitis to the patients that underwent cholecystectomy for another diagnosis. Three hundred fifty-three patients suffered from acute cholecystitis. Sixteen of these 353 patients (4.5%) developed complications compared to 24 (1.9%) of the non-acute patients (*p* = 0.01). Acute cholecystitis patients had a drain placed significantly more often (42.8% vs. 9.1%, *p* < 0.001, *R* 0.38, medium effect size) and had a longer postoperative hospital stay (median of 3 vs 2 days, SD ± 3.6 vs ± 1.8, *p* < 0.001, *R* 0.35, medium effect size). When comparing the preoperative and postoperative blood tests of the patients undergoing CCE for acute cholecystitis and those undergoing CCE for non-acute reasons, the median blood test results were significantly different. However, the effect sizes were low for preoperative and postoperative hemoglobin and total bilirubin. The same was true for preoperative GPT and postoperative leukocytes. The difference between the preoperative leukocytes in these two groups had a medium effect size. While nearly all of these laboratory tests were statistically different, most median values were within the normal range (!) in both groups. For details, see Table 4.

**Table 4:** Compares the postoperative lab results, drains, and length of stay between the patients suffering from acute cholecystitis to those that underwent cholecystectomy for other reasons

Acute vs. non-acute cholecystitis			
	Acute cholecystitis	Other	*p* value
*n* (%)	353 (22.1%)	1233 (77.9%)	
Leukocytes preop µl (SD)	11,020 (± 5650)	6980 (± 3560)	0.00
Leukocytes POD 1/µl (SD)	10,400 (± 4560)	8870 (± 3480)	0.00
Hemoglobin preop, mg/dl (SD)	13.3 (± 1.8)	13.7 (± 1.5)	0.00
Hemoglobin POD 1 mg/dl (SD)	12.1 (± 1.7)	12.8 (± 1.5)	0.00
Bilirubin preop mg/dl (SD)	0.8 (± 1.0)	0.6 (± 0.7)	0.00
Bilirubin POD 1 mg/dl (SD)	0.7 (± 0.9)	0.8 (± 0.7)	0.00
GPT preop U/l (SD)	35 (± 101)	27 (± 92)	0.02
GPT POD 1 U/l (SD)	53 (± 64)	51 (± 91)	0.27
Drains	151 (42.8%)	113 (9.1%)	0.00
Length of stay in day (SD)	3 (± 3.6)	2 (± 1.8)	0.00

### Acute cholecystitis

Within the group of acute cholecystitis, both the preoperative total bilirubin and the total bilirubin on POD 1 were different between the patients that develop postoperative bile duct occlusion (due to stone or intraoperative injury). Six of the 353 patients were diagnosed with a biliary occlusion postoperatively. Bilirubin was available in 327 patients preoperative and 318 patients on POD 1. Patients with biliary occlusion had a significantly higher total bilirubin both preoperative (median of 1.4 mg/dl ± 3.3 vs 0.8 ± 0.9, *p* = 0.049, *R* 0.11, low effect size) and postoperative (median of 3.8 mg/dl ± 2.3 vs 0.7 ± 0.7, *p *< 0.001, *R* 0.17, low effect size). Postoperative hyperbilirubinemia had a sensitivity of 83.3%, specificity of 79.6% a positive predictive value of 7.4%, and a negative predictive value of 99.6% for predicting bile duct occlusion. There was no difference between perioperative hemoglobin, white cell count, or GPT with regard to the other complications.

### Elective cholecystectomy

Within the group of elective cholecystectomies, both the preoperative total bilirubin and the total bilirubin on POD 1 are different between the patients that develop postoperative bile duct occlusion (due to stone or intraoperative injury). Of the 1227 patients, 8 were diagnosed with a biliary occlusion postoperatively. Bilirubin was available in 1180 patients preoperative and 1167 patients on POD 1. Patients with biliary occlusion had a significantly higher postoperative total bilirubin, however with a very minor effect (median of 0.9 mg/dl ± 1.3 vs 0.6 ± 0.7, *p *= 0.048, *R* 0.04, low effect size). Postoperative hyperbilirubinemia had a sensitivity of 55.6%, specificity of 76.6%, a positive predictive value of 1.8%, and a negative predictive value of 99.6% for predicting bile duct occlusion. We did not see a significant difference between the perioperative hemoglobin, white cell count, or GPT with regard to the other complications.

## Discussion

While laparoscopic cholecystectomies are a common procedure and complications are rare, those who develop complications are often at severe risk. Given the increasing trend towards performing laparoscopic cholecystectomy as day case procedures, we are in need of early prognostic tools to predict complications. This is complicated by the fact that both bile leaks and choledocholithiasis can present even weeks after discharge [10]. In our patient cohort, seven patients were readmitted after an uneventful hospital stay and scheduled discharge on POD 2 with severe complications at a later date. The goal of this study was to evaluate the potential of postoperative blood tests to predict complications. Postoperative labs and ultrasound are the standard of postoperative care in order to identify early postoperative complications in most hospitals around the globe. Our center discontinued routine ultrasound in 2015 after finding a high number of false positives combined with a low sensitivity in discovering clinically relevant complications. To our knowledge, routine ultrasounds are still common practice in many hospitals, but data is pointing more and more towards a more selective use [4, 6]. There are no randomized controlled studies that compared the outcome of patients that do not receive postoperative blood tests with patients that do not. A retrospective study from Israel reported on their department’s results on less liberal use postoperative blood tests. Ben-Ishay et al. described a retrospective cohort of 532 elective cholecystectomies where blood was only drawn if the attending deemed it necessary. In their cohort, older patients and those patients, whose CCE took longer (a surrogate for more complex procedure?), received blood tests more frequently. They reported that there was no increased rate in complications in the patients that had no postoperative blood tests [11]. Recently Fischer et al. proposed a risk score on whom to test postoperatively with the ultimate goal of supporting the introduction of CCE as a day case in Germany [12]. They defined several perioperative risk factors that assist the surgeon in her/his decision on whom can be discharged early after CCE without additional testing. They came to the conclusion that about 80% of post-op labs they drew could have been avoided in hindsight. While this is a very interesting approach, their proposed scoring system needs to be validated in a larger cohort, since it was only tested retrospectively in 100 patients.

Another factor in the decision-making process is the emergence of medical litigation cases. Failure to predict or detect complications early has become a new driver for postoperative diagnostics [13, 14]. Post-op labs are the most common diagnostic tool besides clinical examination. Unfortunately there is very limited data on the benefits of this practice. The largest prospective study on the predictive value of preoperative blood tests described alkaline phosphatase and bilirubin to be the most valuable parameters predicting postoperative choledocholithiasis. However, the study also suffered from a large portion of false positives [7]. They also did not report on postoperative blood test.

Our study found a high rate of preoperative hyperbilirubinemia especially in patients suffering from acute cholecystitis. In our overall analysis, both preoperative and postoperative hyperbilirubinemia were predicting postoperative (or undetected) CDL similarly well. However, this effect was reverted when we excluded patients with preexisting hyperbilirubinemia. In a subgroup analysis of the cases of acute cholecystitis, we did see a small predictive value of postoperative hyperbilirubinemia. There appeared to be little to no benefit however of postoperative blood tests in elective procedures.

Several studies have investigated the natural course of post-op liver enzymes and compared laparoscopic cholecystectomy to open cholecystectomy [15–17]. In laparoscopic surgery, there is a trend towards a higher elevation in transaminases with spontaneous return after 3–7 days. Most authors attribute this to the capnoperitoneum which has been also studied in an animal model [18]. We measure GPT/ALT levels on POD1 mainly to detect major vascular injury. In this study period, no major vascular injury was documented, and thus, there is only minor insight we can offer to understand the role of GPT/ALT levels post-hepatic artery ligation in cholecystectomy. Even without any major injuries, the highest GPT/ALT we detected on POD 1 was 30 times above the upper limit (1509 U/l). The patient did not develop a clinically relevant complication.

Postoperative labs on POD 1 did not predict complications well enough to warrant regular testing for every patient. Some patients that were discharged in good condition on POD 2 were readmitted with major complications, while most patients with abnormal blood tests on POD 1 had inconspicuous tests on POD 2 and were discharged without developing further complications. Hemoglobin levels on POD 1 decreased by > 3 mg/dl in over 40 patients. However, we discovered only eleven episodes of postoperative hemorrhage. The two patients that presented with life-threatening acute bleeding were detected due to clinical deterioration, not routine blood tests. The high rate of hyperbilirubinemia on POD 1 triggered several repeat blood tests as well as imaging studies.

Elevation of bilirubin on POD 1 is a common finding most likely caused by the acute phase reaction. This was more common in patients that did not suffer from acute cholecystitis. Patients with acute cholecystitis frequently presented with hyperbilirubinemia. Those patients had the highest median of preoperative bilirubin. Their bilirubin levels frequently dropped after source control was achieved during surgery. The only other study evaluating postoperative blood tests after cholecystectomy (done by Pochhammer et al.) describes a cohort of 816 patients that received routine blood tests on POD 2 [4]. They reported on 31 cases of surgical infections as well as 22 cases of CDL. Aside from the lack of benefit of routine postoperative ultrasound, they also described similar limitations when interpreting individual liver enzymes postoperatively with a sensitivity and specificity similar to ours. In order to alleviate this problem, they established cutoffs for bilirubin, aspartate-aminotransferase, and alkaline phosphatase and reported a number needed to monitor in order to detect CDL. Once all these perimeters were above their established best cutoffs, they performed ultrasound to rule our bile duct dilation, followed by ERCP when the duct was dilated. Whether these patients would have developed symptoms in the future, no one knows. While we cannot reproduce their results due to different blood tests and time frames, we agree that the key to a better prediction may be the combination of several individual factors.

Pochhammer et al. furthermore reported on the value of postoperative c-reactive protein which in turn was able to predict infectious complications, when elevated more than 24 × the upper limit on POD 2. They report that an 8 out of 9 patients with a CRP of > 12.3 mg/dl on POD 2 had an infectious complication. The clinical impact of this appears to be rather minor given the 28 uncomplicated superficial wound infections that they encountered in their study. While CRP has been established as predictor for anastomotic leak in colorectal surgery [19], it is true benefit in cholecystectomy needs to be studied further before we will include it in our protocol.

Ultimately it appears that every department needs to weigh strengths and weaknesses of postoperative blood tests when developing their postoperative protocol. The main benefit we did discover was the high negative predictive value of early routine blood testing. Patients with unremarkable intraoperative course and inconspicuous presentation on POD 1 likely do not benefit from routine blood testing. Hyperbilirubinemia and drops in hemoglobin should not routinely trigger additional testing in clinically healthy subjects. In societies, where physicians are at increased risk of medical litigation, early normal blood tests on POD 1 may support the decision to discharge patients early. When it comes to postoperative leukocytosis and hyperbilirubinemia, blood tests on POD 2 may lower the risk of false positives caused by the acute phase reaction at the cost of potentially delaying patient discharge.

## Strength and limitations

The strength of our study is the large sample size of an academic teaching hospital with a focus on laparoscopic surgery and a high volume hepatobiliary unit. To our knowledge, we presented by far the largest cohort of laparoscopic cholecystectomies focusing on the value of postoperative blood work. All patients underwent a standardized postoperative protocol and were registered in our prospective database. Protocol adherence was satisfying. This collective of patients resembles a spectrum of patients from a middle European region. The median age in our study appears to be higher, and the patients’ median BMI may be lower than in comparable studies from different regions. This may be due to a referral bias. Furthermore, the study design provides only retrospective data, and the number of individual complications is low, which compromises statistical analysis with logistic regression. Our hospital is the only provider of gallbladder intervention and surgery in our district. While patients are encouraged to see our department with postoperative problems, there is a risk for unnoticed drop-outs or presentations to other hospitals that are not recorded by our in house data, since we did not get into contact with the patients systematically during data collection.

## Conclusion

Laparoscopic cholecystectomy is a highly standardized surgical procedure. There is limited data on the benefits of postoperative diagnostics, especially in uncomplicated cases. This retrospective cohort analysis of a tertiary academic teaching hospital was designed to show the limited value of early postoperative bloodwork to detect complications after routine cholecystectomy. In our patient collective, we were able to demonstrate how low sensitivity and positive predictive values of postoperative blood tests are. Likely, the highest benefit offered by postoperative blood tests is the negative predictive value once they are within the normal range. While randomized, controlled trials are needed, it appears that both routine postoperative ultrasound and blood tests in asymptomatic patients lead to unnecessary additional testing and costs. While detecting clinically irrelevant findings, both fail to reliably predict true complications. The intraoperative placement of drains and postoperative diagnostic testing should be done at the discretion of the operating surgeon based on specific intraoperative findings (e.g., severe inflammation, difficult to close cystic duct, diffuse bleeding) and postoperative clinical examination of the patient (e.g., local peritonism).

## Data Availability

All data and material can be provided on further request.

## Abbreviations

ALT: Alanine-amino-transferase
AUC: Area under the curve
CBC: Complete blood count
CBD: Common bile duct
CCE: Cholecystectomy
CDL: Choledocholithiasis
CI: Confidence interval (95%)
CRP: C-reactive protein
ERCP: Endoscopic retrograde cholangio-pancreaticography
HJ: Hepatico-jejunostomy
GPT: Glutamate-pyruvate-transaminase
POD: Postoperative day
SD: Standard deviation
